# The effectiveness of marine based fatty acid compound (PCSO-524) and firocoxib in the treatment of canine osteoarthritis

**DOI:** 10.1186/s12917-019-2110-7

**Published:** 2019-10-17

**Authors:** Monchanok Vijarnsorn, Irin Kwananocha, Narudee Kashemsant, Thitichai Jarudecha, Chalermpol Lekcharoensuk, Brian Beale, Bruno Peirone, B. Duncan X. Lascelles

**Affiliations:** 1 Department of Companion Animals Clinical Sciences. Faculty of Veterinary Medicine, Kasetsart University, Bangkok, 10900 Thailand; 2 Department of Physiology, Faculty of Veterinary Medicine, Kasetsart University, Bangkok, 10900 Thailand; 3 Gulf Coast Veterinary Specialists, Houston, TX USA; 4 Dipartimento di Scienze Veterinarie, Facoltà di Medicina Veterinaria, Largo, P. Braccini, 2-5, 10095 Grugliasco (To), Italy; 5 Translational Research in Pain Program, Comparative Pain Research and Education Centre, Department of Clinical Sciences, College of Veterinary Medicine, North Carolina State University, Raleigh, NC USA; 6 Center for Translational Pain Research, Department of Anesthesiology, Duke University, Durham, NC USA; 7 Thurston Arthritis Center, UNC, Chapel Hill, NC USA

**Keywords:** Osteoarthritis, Treatment, PCSO-524, NSAIDs, Firocoxib, Gait analysis, Dog

## Abstract

**Background:**

NSAIDs are accepted as the most predictably efficacious medical treatment of the clinical signs of osteoarthritis (OA). The marine-based fatty-acid compound PCSO-524 has been proposed as an adjunctive treatment for canine OA, however benefits of this agent is still controversial. The purpose of this study was to evaluate and compare the effectiveness of PCSO-524 combined with the NSAID firocoxib using force plate gait analysis, orthopedic assessment score (OAS) and canine brief pain inventory score (CBPI) in dogs with OA.

A prospective, randomized, double-blinded study was conducted. Seventy-nine dogs that had hip and/or stifle OA were assigned randomly into three treatment groups: firocoxib, PCSO-524 and combination of firocoxib and PCSO-524, orally for 4 weeks. Peak vertical force (PVF, expressed as a percentage of bodyweight), OAS, CBPI, serum prostaglandin E_2_ concentration, hematology and blood chemistry values were evaluated before treatment (Day0), as well as at the second (Day14) and fourth week (Day28) during treatment.

**Results:**

Within group analysis revealed significant increases in PVF over the 4-week treatment period for firocoxib, PCSO-524 and the combination (*p* < 0.05). Mean increases in PVF were 3.25 ± 4.13, 2.01 ± 3.86, 4.11 ± 4.69%BW (mean ± SD) respectively. The OAS showed non-significant change in all treatment groups. There were significant decreases in CBPI pain severity score (PSS) and CBPI pain interference scores (PIS) within some groups over time, however no significant differences were found between the groups. Significantly decreased serum PGE_2_ concentration (*p* < 0.05) was found in the combination group. Significant increases in BUN and creatinine (*p* < 0.05) compared to pre-treatment values were found in the firocoxib and combination groups but not in the PCSO-524 group at day28, but all values in all dogs remained within the normal ranges.

**Conclusions:**

The results of this study suggested combination of both PCSO-524 and firocoxib is more effective in alleviation of inflammation and improvement of weight bearing ability when compared to the uses of either PCSO-524 or firocoxib alone. Further clinical studies are needed to confirm this, and to determine if there is any benefit of PCSO-524 over placebo.

## Background

Osteoarthritis (OA) is a chronic progressive disease that adversely affects quality of life of animals [1, 2]. Osteoarthritis leads to structural and function alterations of the affected joint [3] through articular cartilage deterioration, synovitis, osteophyte formation and joint capsule inflammation and thickening [1, 4], although arguably the most important aspect is the pain that can be associated with the condition. Pain results in both local (e.g. within the limb) and distant (other parts of the body) deterioration of the musculoskeletal system as a result of decreased and altered mobility. Additionally, the ongoing nociceptive input into the central nervous system results in somatosensory system functional changes and central sensitization [5], which contributes to the overall perception of pain.

Non-steroidal anti-inflammatory drugs (NSAIDs) are widely used for the amelioration of OA pain in dogs [1, 6, 7]. They exert their effect via inhibition of the cyclooxygenase (COX) enzymes, blocking the synthesis of prostanoids, including prostaglandin E_2_ (PGE_2_), a potent inflammatory mediator [7]. As a result of blocking the production of prostanoids, NSAIDs can cause undersirable side effects, although these likely occur at a low incidence. However the true incidence of adverse events is currently unknown [8]. Regardless, because of fears of side effects, and because not all dogs respond sufficiently to NSAIDs alone [9], there is interest in other drug and non-drug approaches.

The marine based fatty acid compound, PCSO-524 is a rich source of long-chain polyunsaturated omega-3 fatty acids (omega-3) extracted from the New Zealand green-lipped mussel (*Perna canaliculus*) using the supercritical carbon dioxide method. This marine based fatty acid product contains a numerous sterol esters, sterols, polar lipids, triglycerides and at least 91 different fatty acids including eicosapentaenoic acid (EPA), docosahexaenoic acid (DHA) and eicosatetraenoic acid (ETA) [10]. This unique combination of multiple omega 3 fatty acids has been shown to have anti-inflammatory effects through the reduction of leukotriene and prostaglandin production in lipoxygenase (LOX) and cyclooxygenase (COX) pathways [11, 12]. In a previous clinical study, PCSO-524 administration resulted in significant improvement of dogs with OA, as did the administration of carprofen, and the combination of PCSO-524 and carprofen, but no improvement was seen with glucosamine/chondroitin sulfate [13]. Intergroup differences were not significant, but these data suggested further investigation of PCSO-524 was warranted.

The current study further investigated the effectiveness PCSO-524 by comparing the administration of firocoxib, PCSO-524 and firocoxib/PCSO-524 combination as treatments of canine OA pain, using the primary outcome measure of objective force plate gait analysis. We hypothesized that the combination of PCSO-524 and firocoxib would result in a superior therapeutic effect when compared to the use of firocoxib or PCSO-524 alone.

## Results

### Animals

Owners of 119 dogs volunteered to participate in the study. After telephone interview and/or physical examination, 82 dogs met the inclusion criteria and were enrolled into the study. During the study, 3 dogs dropped out of the study due to cranial cruciate ligament rupture (*n* = 1), myasthenia gravis becoming clinically evident (*n* = 1) and a vehicular accident (*n* = 1). At the end of the study, 79 dogs were used in the statistical analyses: 24 dogs in firocoxib group, 27 dogs in PCSO-524 group and 28 dogs in combination group. The breeds included Golden Retrievers (*n* = 43), Labrador Retrievers (*n* = 23), Siberian Husky (*n* = 5), crossbreed (*n* = 3) Thai Bangkeaw (*n* = 1) Cane Corso (*n* = 1) and American Pitbull (*n* = 3). The average age and weight were 4.7 ± 2.99 years old and 36.15 ± 7.18 kg (mean ± SD) respectively. Fifty-seven dogs were classified as having mild to moderate OA whereas 22 dogs were graded as having severe osteoarthritis. Among these 79 canine patients, 77 dogs had bilateral hind limb lameness associated with OA of the hip and/or stifle (Table 1).

**Table 1:** Demographic variables at day 0 for the 3 treatment groups and comparison across groups

Variable	Firocoxib	PCSO-524	Combination	*P* value
Number of dogs	24	27	28	
Severity of clinical sign				0.929
- Mild to moderate	18	19	20	
- Severe	6	8	8	
Body weight ± SD (kg)	34.82 ± 6.62	37.53 ± 8.55	35.77 ± 6.13	0.394
Age ± SD (years)	4.46 ± 2.89	5.44 ± 3.18	4.18 ± 2.88	0.267
Body condition score				0.284
- 1/5	0	0	1	
- 2/5	1	0	0	
- 3/5	12	11	11	
- 4/5	10	9	13	
- 5/5	1	7	3	
Breed				0.457
- Golden retriever	13	13	17	
- Labrador retriever	6	10	7	
- Siberian husky	3	0	2	
- Crossbreed	2	1	0	
- Bangkeaw	0	1	0	
- Cane corso	0	1	0	
- American pitbull	0	1	2	
Sex				0.926
- Male	9	11	12	
- Female	15	16	16	
Joint affected				0.171
- Hip	20	20	26	
- Hip and Stifle	4	7	2	
Side of affected joint				0.582
- Unilateral	1	0	1	
- Bilateral	23	27	27	
Side of affected limb				0.899
- Left	14	17	16	
- Right	10	10	12	
Duration of signs (months)	13.46 ± 13.82	14.07 ± 10.84	12.75 ± 13.19	0.927
OAS	5.42 ± 2.70	6.59 ± 2.99	5.43 ± 2.28	0.188
CBPI	1.62 ± 1.29	2.23 ± 1.97	1.62 ± 1.65	0.310
PVF (%BW)	64.79 ± 5.98	62.01 ± 6.85	59.97 ± 9.38	0.081

At the start of the study, severity of clinical signs (*p* = 0.929), weight (*p* = 0.394), age (*p* = 0.267), breed (*p* = 0.457), sex (*p* = 0.926), joints affected and side (*p* = 0.171 and 0.582), duration of lameness (*p* = 0.927), OAS (*p* = 0.188), CBPI (*p* = 0.311) and PVF (*p* = 0.082) were not significantly different among the 3 treatment groups.

### Force plate gait analysis; peak vertical force

Repeated measures analysis demonstrated a non-significant treatment effect on the PVF values (*p* = 0.069) among the three treatment groups, but there was a significant effect of time (*p* < 0.05). By the day 14, PVF values of the firocoxib, PCSO-524 and combination groups were significantly greater than pre-treatment values (*p* < 0.05) with mean ± SD change 3.03 ± 0.67, 1.82 ± 3.22 and 2.74 ± 4.41%BW respectively. After 4 weeks of treatment, PVF values of the firocoxib, PCSO-524 and combination groups were significantly greater than their pre-treatment values (*p* < 0.05) with the mean ± SD changes of 3.25 ± 4.13, 2.01 ± 3.86, 4.11 ± 4.69%BW respectively. There were no significant differences between week two and week four for PVF (Table 2). There were no significant differences of mean velocity of dogs among 3 treatment groups at any time point.

**Table 2:** PVF values at pre-treatment (day0), day14 and day28 for each group, and the change from baseline

	Time	Firocoxib	PCSO-524	Combination	*P*-value^*^
PVF (%BW)	Day 0 (PVF)	64.79 ± 5.98 ^a^	62.01 ± 6.85 ^a^	59.97 ± 9.38 ^a^	0.069
	Day 14 (PVF) Mean change±SD	67.82 ± 6.70 ^b^ 3.03 ± 4.67	63.82 ± 6.15 ^b^ 1.82 ± 3.22	62.71 ± 9.15 ^b^ 2.74 ± 4.41	
	Day 28 (PVF) Mean change±SD	68.05 ± 6.29 ^b^ 3.25 ± 4.13	64.01 ± 6.52 ^b^ 2.01 ± 3.86	64.08 ± 9.99 ^b^ 4.11 ± 4.69	

*SD* standard deviation

^a, b^*p*-value < 0.05 from Tukey’s Studentized Range within each treatment

^*^p-value from repeated measurement analysis for 3 treatments

### Orthopedic assessment score

There was no significant treatment (group) effect (*p* = 0.156) and no significant within-group change over time (*p* > 0.05) (Table 3).

**Table 3:** OAS values (mean ± SD) at pre-treatment (day 0), day 14 and day 28 for each treatment group

	Time	Firocoxib	PCSO-524	Combination	*P*-value^*^
OAS	Day 0 (point)	5.42 ± 2.70 ^a^	6.59 ± 2.99 ^a^	5.43 ± 2.28 ^a^	0.1559
	Day 14 (point)	6.13 ± 2.44 ^a^	6.59 ± 2.96 ^a^	5.39 ± 2.57 ^a^	
	Day 28 (point)	5.79 ± 2.60 ^a^	6.93 ± 1.94 ^a^	5.71 ± 2.66 ^a^	

*SD* standard deviation

^a^*p*-value < 0.05 from Tukey’s Studentized Range within each treatment

^*^p-value from repeated measurement analysis for 3 treatment

### Canine brief pain inventory score

There were no significant treatment effects for pain severity (PSS) or pain interference scores (PIS). The within-group comparisons by the use of the Turkey’s studentized range showed significant decreases in the PSS at week four compared with pre-treatment values in the PCSO-524 group (*p* < 0.05) (Table 4). Significant decreases in the PIS at week four compared with the pre-treatment values were demonstrated in the Firocoxib and PCSO-524 groups (*p* < 0.05) (Table 5).

**Table 4:** PSS values (mean ± SD) at pre-treatment (day 0), day 14 and day 28 for each group

	Time	Firocoxib	PCSO-524	Combination	*P*-value^*^
PSS	Day 0 (point)	1.89 ± 2.02 ^a^	2.67 ± 2.58 ^a^	2.04 ± 2.40 ^a^	0.4834
	Day 14 (point)	1.78 ± 1.82 ^a^	2.40 ± 2.36 ^ab^	1.63 ± 1.73 ^a^	
	Day 28 (point)	1.88 ± 1.97 ^a^	1.56 ± 1.64 ^b^	1.27 ± 1.98 ^a^	

*SD* standard deviation

^a, b^*p*-value < 0.05 from Tukey’s Studentized Range within each treatment

^*^p-value from repeated measurement analysis for 3 treatments

**Table 5:** PIS values (mean ± SD) at pre-treatment (day 0), day 14 and day 28 for each group

	Time	Firocoxib	PCSO-524	Combination	*P*-value^*^
PIS	Day 0 (point)	2.10 ± 1.71 ^a^	2.95 ± 3.01 ^a^	1.92 ± 2.17 ^a^	0.2830
	Day 14 (point)	1.72 ± 1.96 ^ab^	2.54 ± 2.75 ^ab^	1.68 ± 2.12 ^a^	
	Day 28 (point)	1.27 ± 1.20 ^b^	1.67 ± 1.63 ^b^	1.30 ± 2.06 ^a^	

*SD* standard deviation

^a, b^*p*-value < 0.05 from Tukey’s Studentized Range within each treatment

^*^p-value from repeated measurement analysis for 3 treatments

### Correlation between PVF and OAS

The Pearson correlation demonstrated negative correlation between PVF and OAS with the coefficients of − 0.259 (*p* < 0.001). Similar negative correlations were found between PVF and PSS or PIS with the coefficients of − 0.245 (*p* < 0.002) and − 0.328 (*p* < 0.001) respectively.

### Serum PGE_2_ concentration

The mean and standard error before and after adjusted baseline of PGE_2_ at week 0, week 2 and week 4 were showed in Tables 6 and 7 respectively. The exploratory data analysis showed a significantly higher baseline PGE_2_ concentration in PCSO-524 group than the others (*p* = 0.019). An ANCOVA approach was performed to adjust the pre-treatment values to 1815.795 ± 0.00 pg/mL (mean ± SE) and use their pretreatment values as covariates. Repeated measures analyses of covariance showed there was no significant treatment effect on PGE_2_ values (*p* = 0.639), but there was a significant effect of time (*p* < 0.05). The within group comparisons revealed there were no significant changes in serum PGE_2_ concentration for the firocoxib and PCSO-524 groups over the 4 weeks of study. However, serum PGE_2_ decreased significantly in the combination group (*p* < 0.05). The Bonferroni test demonstrated significant reductions of serum PGE_2_ concentration between week 0 and week 4 (*p* = 0.036) as well as between week 2 and week 4 (*p* = 0.039) in the combination group.

**Table 6:** PGE_2_ level (Mean ± SE) at pre-treatment (day 0), day 14 and day 28

	Time	Firocoxib	PCSO-524	Combination	*P*-value^*^
PGE_2_ (pg/mL)	Day 0	1725.57 ± 118.28^a^	1992.62 ± 70.80^b^	1722.62 ± 64.73^a^	0.019
	Day 14	1667.30 ± 126.76	1887.28 ± 77.76	1735.57 ± 90.21	
	Day 28	1685.67 ± 86.55	1868.96 ± 69.39	1556.45 ± 116.38	
	N	24	27	28	

*SE* standard error

^a, b^Multiple comparison using Bonferroni test with *p*-value < 0.05

^*^p-value from repeated measurement analysis for 3 treatments

**Table 7:** PGE_2_ level after adjusted baseline (Mean ± SE) at pre-treatment (day 0), day 14 and day 28

	Time	Firocoxib	PCSO-524	Combination	*P*-value ^**^
Adjusted PGE_2_^a^ (pg/mL**)**	Day 0	1815.795 ± 0.00	1815.795 ± 0.00	1815.795 ± 0.00^a^	0.639
	Day 14	1721.642 ± 88.889	1780.773 ± 85.645	1791.691 ± 82.430^a^	
	Day 28	1717.807 ± 94.695	1805.974 ± 91.239	1589.641 ± 87.814^b^	

^**^p-value from repeated measurement ANCOVA for 3 treatments

^a, b^*p*-value < 0.05 from multiple comparison within each treatment using Bonferroni test

^a^Covariates appearing in the model are evaluated at PGE_2_ of every group at week 0 = 1815.795 pg/mL

### Hematology and blood chemistry values

The clinical laboratory values of all dogs were within normal limits during the study period of 4 weeks. However, repeated measurement analyses demonstrated a significant treatment effect on blood urea nitrogen (BUN) (*p* < 0.001) and creatinine (*p* = 0.013) but not on pack cell volume (PCV), white blood cell count (WBC), platelet count, alanine aminotransferase (ALT), alkaline phosphatase (ALK) and the A:G ratio.

The within-group comparisons using the Turkey’s Studentized Range showed a significant increase in BUN at day 14 and day 28 when compared with pre-treatment values in the firocoxib and combination groups (*p* < 0.05). The creatinine values at day 14 and day 28 were significantly increased when compared with pre-treatment values in the combination group (*p* < 0.05). A significant increase in creatinine between pre-treatment and day 28 was seen in the firocoxib group (*p* < 0.05). However, BUN, creatinine values and urine specific gravity of all dogs in this study remained within normal range at all time points.

## Discussion

The results of this study indicate that there appear to be benefits of both firocoxib and PCSO-524 for the treatment of the clinical signs associated with canine OA. Although significant differences between treatment groups was not shown, the change in peak vertical force (PVF) in the combination group was numerically superior to the other two groups. This may indicate beneficial effects of using PCSO-524 in combination with firocoxib, but further data, including a placebo-treated group, are needed to explore this. It is possible that the NSAIDs and PCSO-524 may exert their effects in a common pathway of arachidonic acid, and if there were a real additive benefit, it may be because both agents may work synergistically to alleviate joint inflammation and pain.

Force plate gait analysis measurement of ground reaction forces is an objective method to gauge limb function and musculoskeletal pain in dogs with appendicular joint OA. It is considered to provide an unbiased and accurate assessment. However, without a placebo treatment group, it is impossible to know if other external factors influenced the dogs in a way that may have resulted in improved limb use. For example, if the owners felt optimistic from knowing their dog was going to receive at least one putative treatment, this may have influenced how their pet felt, which may have caused real changes in pain – as was found recently in work in cats with OA receiving placebo [14]. The increases in PVF seen in this study were modest, and without a placebo group, it is impossible to know if these changes in PVF reflected real positive treatment effects. However, a recent study using a cross-over design, where every dog received tramadol or carprofen (or placebo) during the blinded study period, found no change in PVF over a 10-day period with the administration of placebo. Another aspect that may have influenced the PVF values is the fact that there was no required minimum difference in PVF between the hindlimbs. Our inclusion criteria allowed for bilateral lameness, and the index limb was the limb with the lowest PVF. This approach to inclusion criteria increases the rate of recruitment and enrollment, but likely decreases the expected change in PVF that will be seen with a successful treatment. Again, this indicates that a placebo group is needed in future studies. On balance, we conclude that this study has documented significant changes in PVF over time in dogs receiving firocoxib, PCSO-524 or the combination of both therapies. The changes in PVF, although relatively modest are similar to other studies. Brown et al. [15] found a mean improvement in PVF of 3.2 (±SD of 0.8), which is very similar to the improvement found in this study (PVF improvement in firocoxib treated dogs of 3.03 ± 4.67 at day 14, and the starting point for limb use (as measured by PVF) was similar in both studies. The mean PIS for the dogs in this study at day 0 was 2.5, compared to 4.8 in previous studies run by the author [16, 17]. This suggests the dogs in the present study had a low level of impairment, although direct comparison of PIS values across these studies may not be appropriate as the CBPI instrument was translated into Thai by bilingual language instructor. Indeed, the change over 14 days in the CBPI scores seen in this study appeared to be much less than in the Brown et al. study (2013), and collectively suggest the translation of the CBPI into Thai resulted in an instrument that did not function as well as when administered in the language it was originally constructed in. The subjective assessment by veterinarians (OAS), PSS and PIS yielded a low negative correlations with PVF. These results are similar to previous work comparing PVF to the CBPI [15, 18]. As has been described previously, [15, 18] the subjective assessments are likely measuring factors other than just pure limb use that is measured by using force plates.

The serum PGE_2_ concentrations found in the dogs in this study were much higher than have been described in normal dogs [19]. All other work describing the effects of anti-inflammatories on PGE_2_ production has used ex-vivo analysis [20]. There was an approximately 10% reduction in PGE2 in the combination group, and this may be clinically meaningful. More research is needed to fully understand the implications of the results seen in our study.

The significant increases in BUN and creatinine levels were found in the firocoxib and combination group but not the PCSO-524 group after 2 weeks of treatment. It appears that the groups receiving the NSAID firocoxib showed increases in BUN and creatinine, and this is not inconsistent with the potential side effects of NSAIDs such as firocoxib [8]. Indeed, in one study that evaluated blood work in 33 dogs (> 7 years old, with osteoarthritis) receiving firocoxib for 90 days found that both BUN and creatinine were significantly increased at the end of the study compared to the start [21]. However, the BUN and creatinine values remained within normal range, [21] similar to the dogs in the present study.

The goals of OA treatment are to reduce pain and inflammation, to prevent or slow down degeneration of the cartilage and to support or restore joint function and overall mobility. To achieve these treatment goals, a multimodal approach to the management of OA, has been advocated [22]. In this respect, the results of this study support the approach of using PCSO-524 in combination with an NSAID to help mitigate clinical signs. However, an important limitation of this study was the lack of a placebo group, and definite conclusions about the effectiveness of PCSO-524, firocoxib or the combination cannot be made until the results are compared to a placebo group.

The major limitations of this study are the lack of a placebo group, and the relatively short duration of administration of test medications. Future studies should include a placebo group and evaluate responses over longer periods of time.

## Conclusion

Although significant differences between groups were not detected in this study, an increased PVF over the 28 days was seen in all treatment groups, with the numerically greatest change demonstrated in the combination group. This may imply beneficial effects of PCSO-524 in combination with NSAIDs. Further work should be performed to confirm this, and evaluate the effects of PCSO-524 alone and in combination with NSAIDs, and against placebo.

## Methods

### Study design

The study was designed as a prospective, block-randomized, double-blinded clinical trial in client owned dogs, and was conducted at the Kasetsart University Veterinary Teaching Hospital, Thailand. The dogs remained under the care of their owners during the study, and after the study. The study protocol was approved by the Institutional Animal Care and Use Committee of the Faculty of Veterinary Medicine, Kasetsart University (ACKU61-VET-032). Informed, written consent was obtained from all owners prior to their dogs being entered in the study.

### Animals

Dogs of either sex, any breed, at least 1 year old, with a body weight of at least 20 kg were eligible to be enrolled into the study. All dogs included in the study were required to have owner reported disability and clinical signs of chronic OA of hip and/or stifle joint (hindlimb lameness and joint pain) and radiographic evidence of OA. Dogs were required to have hematology and blood chemistry values within normal limits. Exclusion criteria were dogs with suspected forelimb OA; dogs with lameness that appeared to be primarily due to stifle instability; dogs that would not trot across the force plate for any reason; dogs with a cruciate rupture with the previous 6 months; dogs with clinically detectable neurological deficits; dogs with history of orthopedic surgery within the preceding 8 months; dogs with clinically detectable systemic diseases; and pregnant or lactating bitches. Prior to starting the study, dogs were required to be not receiving analgesics. A two-week washout period for NSAIDs and oral nutraceuticals was required, and 4 weeks for corticosteroids and injectable sodium-pentosan polysulfate. During the study, no other pain-modified therapies were permitted. As described later, the index limb was defined as the hindlimb with the lowest peak vertical force at screening.

### Randomization and blinding procedures

Enrolled dogs were classified into two categories (mild/moderate signs and severe signs) according to the severity of their OA based on a veterinary examination. All examinations were performed by a single veterinarian (MV). The severe category was defined as dogs with a lameness score ≥ 3 (obvious lameness when walking and trotting) and joint pain score (hip and/or stifle joints) of 3 (dog vocalizes or becomes aggressive on manipulation), based on scoring system of Moreau et al. (Table 2 in [2]). The remaining dogs were placed in the mild/moderate category. The severity category was used as a blocking factor in the randomization process to ensure the equal distribution of severity in all three treatment groups. Randomization was achieved using proprietary statistical software (SAS University Edition, SAS Institute, Cary, North Carolina, USA). All evaluators and owners were blinded to the treatment assignment. Randomization and drug dispensing were conducted by a veterinarian who was not involved with patient evaluation. Placebos were manufactured to be identical in appearance to the actual firocoxib and PCSO-524 products. The treatment key was concealed until the data were analyzed.

### Drugs and dosing procedures

Dogs were randomly assigned to one of three groups: Group 1 (Firocoxib) received firocoxib (Previcox®, Merial Limited; 5 mg/kg, q24hr PO) and a PCSO-524 placebo (4 capsules/day, q24hr PO) for 28 days. Group 2 (PCSO-524) received PCSO-524 (Antinol®, Pharmalink International Limited; 4 capsules/day, q24hr PO) and firocoxib placebo for 28 days. Group 3 (Combination) received firocoxib (5 mg/kg q24hr PO) and PCSO-524 (4 capsules, q24hr PO) for 28 days. Each Previcox® tablet contained firocoxib 227 mg, and the corresponding placebo tablets contained starch. Placebo tablets were dosed in the same manner as firocoxib tablets would have been administered. Each PCSO-524 capsule contained PCSO-524 50 mg, olive oil 100 mg and d-Alpha-tocopherol 0.225 mg. Each PCSO-524 placebo capsule contained sunflower seed oil 139.5 mg, gelatin 150 bloom 111.3 mg, water 106 mg, glycerin 47.7 mg, soy lecithin 7 mg and annatto oil soluble#03160 3.5 mg.

Each dog and owner visited the hospital for a total of three visits: before treatment, followed by two (day 14) and 4 weeks (day 28) post treatment. It was decided a priori that dogs would be withdrawn from the study if their lameness increased (subjective evaluation by attending veterinarian, MV) at any time during the study, and would be investigated and treated appropriately in the veterinary hospital. Rescue treatment for OA pain would consist of an NSAID with an adjunctive drug such as gabapentin or amantadine.

### Patient evaluations and outcome variables

#### Primary outcome measures

##### Force plate gait analysis; peak vertical force

Ground reaction force values were collected using a biomechanical strain gauge dual, in series, force plates (Model OR6–6; Advanced Mechanical Technology, Watertown, MA), embedded in the middle of a 10-m-long walkway. The study dogs were trotted across the force plates by a single handler. Velocity was measured by three laser sensors mounted 2 m apart. The velocity was limited to a range of 1.8–2.2 m/s. A video camera (Nikon 1 J5, Nikon Corporation, Japan) recorded each pass to allow the confirmation of the proper foot strikes and trotting gait. The valid trial was defined as the forelimb followed by the ipsilateral hind limb as it struck the force plate when the dog trotted.

The signals from the dual force plates were acquired and processed through the use of a proprietary software (Cortex 4.0; Motion Analysis Corporation, Santa Rosa, CA) to measure the peak vertical force (PVF). The mean value of PVF (expressed as a percentage of bodyweight, %BW) of each dog for each evaluation time point was derived from the average PVF of the first five valid trials. PVF was normalized to body weight, and expressed as a percentage of total body weight (%BW). The hind limb with the smallest value of PVF was denoted as the index limb at the initial evaluation and the index limb was followed for improvement of limb function during the study period.

#### Secondary outcome measures

##### Orthopedic assessment scores (OAS)

The orthopedic scoring system used in this study has been described previously by Moreau et al. [2]. A composite score was used which comprised the added scores in each of 3 categories: lameness score, articular mobility score and articular pain score. Orthopedic examinations were performed by the same veterinary orthopedic surgeon for all cases throughout the study.

##### Canine brief pain inventory score (CBPI)

At each visit, owners completed a paper copy of the canine brief pain inventory questionnaire (CBPI) [23]. The original CBPI was translated into Thai by a bilingual expert and administered by the same veterinarian for every visit to ensure similar environmental factors during data collection. Average pain severity and pain interference scores for each dog, at each time point were calculated after the hard copies were transcribed into a spreadsheet.

### Analysis of serum prostaglandin E2 concentration

PGE_2_ was analyzed using PGE_2_ assay kit (Parameter™, PharmPak, R&D Systems, Minneapolis, MN) performed at room temperature. The standard curve was prepared as recommended by the manufacturer (0, 39, 78, 156, 313, 635, 1250, and 2500 pg/dl). The 150 μl of either 3 fold diluted samples or standard samples as well as 50 μl of primary antibody were added into the goat anti-mouse PGE2 antibody coated 96 well plate except for the non-specific binding well (NSB well) in which 200 μl of diluents was added instead. Following incubation for 1 h at room temperature on a horizontal orbital microplate shaker at 500 rpm, 50 μl of PGE2 conjugate was added to all wells. The plate was securely covered, incubated for 2 h at room temperature on the shaker and washed 4 times before adding 200 μl of substrate solution to each well. Following 30 min incubation, the optical density of each well was obtained after 100 μl of stopping solution was added, using a microplate reader set to 450 nm with the wavelength correction at 540 or 570 nm.

### Hematology and blood chemistry evaluations

Blood was sampled for complete blood counts and serum biochemical profiles at each of the 3 evaluation times (before treatment, day 14 and day 28). The serum biochemistry evaluations included creatinine, blood urea nitrogen, alkaline phosphatase, alanine aminotransferase, total protein, albumin, the albumin to globulin ratio (A:G ratio).

### Statistical analysis

The sample size was calculated based on the previous work [13] using GPower 3.1.9.2 software (Franz Faul, Universität Kiel, Germany), and based on detecting a difference between the PCSO-524 group and the combination treatment group. We used an expected difference in change in PVF over time of 4.48, with a pooled SD of 3.45, and alpha and beta values of 0.05 and 0.9. This indicated group sizes of 27 would be required. The homogeneity between the experimental groups was evaluated with the Pearson chi-square for breed, sex and severity of OA. Pretreatment values for duration of lameness, age, body weight, PVF, OAS and CBPI pain and CBPI interference were compared between groups using analysis of variance. The data were analyzed using a generalized linear model to assess the treatment effects (SAS University Edition, SAS Institute, Cary, North Carolina, USA). PVF, OAS and CBPI pain and CBPI interference data as well as clinical laboratory parameters were analyzed by repeated-measures analysis of variance by the method of generalized linear model. Each outcome variable was put into the model one at a time. The Tukey’s Studentized Range was used post-hoc to compare differences between time periods. Correlations between PVF and OAS or CBPI were analyzed using Pearson correlation. The effect of the treatment on velocity were tested using generalized linear model. The serum PGE_2_ concentration were analyzed by analysis of covariance (ANCOVA) (SPSS 23, IBM, New York, USA). After the adjustment of the PGE_2_ concentration of all groups using the pretreatment values as covariates, the repeated measures analysis of covariance was performed to test the treatment effect. The Bonferroni correction (where observed *p*-values are multiplied by the number of statistical comparisons performed, the approach used by SPSS) was used for multiple comparisons. For all statistical analyses, the significant level was set at 5% (*P* = 0.05) for the adjusted *p*-value.

## Data Availability

The datasets used and/or analysed during the current study are available from the corresponding author on reasonable request.

## Abbreviations

A:G ratio: Albumin to globulin ratio
ALK: Alkaline phosphatase
ALT: Alanine aminotransferase
BUN: Blood urea nitrogen
BW: Body weight
CBPI: Canine brief pain inventory
NSAIDs: Non-steroidal anti-inflammatory drugs
OA: Osteoarthritis
OAS: Orthopedic assessment score
PCV: Pack cell volume
PIS: Pain interference scores
PO: Per oral
PSS: Pain severity score
PVF: Peak vertical force
WBC: White blood cell
