# Correction to: The effectiveness of marine based fatty acid compound (PCSO-524) and firocoxib in the treatment of canine osteoarthritis

**DOI:** 10.1186/s12917-020-02713-9

**Published:** 2020-12-16

**Authors:** Monchanok Vijarnsorn, Irin Kwananocha, Narudee Kashemsant, Thitichai Jarudecha, Chalermpol Lekcharoensuk, Brian Beale, Bruno Peirone, B. Duncan X. Lascelles

**Affiliations:** 1 Department of Companion Animals Clinical Sciences. Faculty of Veterinary Medicine, Kasetsart University, Bangkok, 10900 Thailand; 2 Department of Physiology, Faculty of Veterinary Medicine, Kasetsart University, Bangkok, 10900 Thailand; 3 Gulf Coast Veterinary Specialists, Houston, TX USA; 4 Dipartimento di Scienze Veterinarie, Facoltà di Medicina Veterinaria, Largo, P. Braccini, 2-5, 10095 Grugliasco (To), Italy; 5 Translational Research in Pain Program, Comparative Pain Research and Education Centre, Department of Clinical Sciences, College of Veterinary Medicine, North Carolina State University, Raleigh, NC USA; 6 Center for Translational Pain Research, Department of Anesthesiology, Duke University, Durham, NC USA; 7 Thurston Arthritis Center, UNC, Chapel Hill, NC USA

**Correction to: BMC Vet Res 15, 349 (2019)**

**https://doi.org/10.1186/s12917-019-2110-7**

The original article [1] omitted competing interests which can be viewed ahead.

**Competing interests**

BDXL has provided, and been renumerated for, continuing educational lectures sponsored by Pharmalink International, including reimbursement of travel expenses. BB has provided, and been renumerated for, continuing educational lectures, appeared in marketing materials and has received research funds and travel expenses from Pharmalink International. The other authors have no other conflicts of interest to declare.
